# Effect of Contrast on Visual Spatial Summation in Different Cell Categories in Cat Primary Visual Cortex

**DOI:** 10.1371/journal.pone.0144403

**Published:** 2015-12-04

**Authors:** Ke Chen, Ai-Min Ding, Xiao-Hua Liang, Li-Peng Zhang, Ling Wang, Xue-Mei Song

**Affiliations:** 1 Key Laboratory for Neuroinformation of Ministry of Education, School of Life Science and Technology, University of Electronic Science and Technology of China, Chengdu 610054, China; 2 Shanghai Institutes of Biological Sciences, Chinese Academy of Sciences, Shanghai 200031, China; University of Toyama, JAPAN

## Abstract

Multiple cell classes have been found in the primary visual cortex, but the relationship between cell types and spatial summation has seldom been studied. Parvalbumin-expressing inhibitory interneurons can be distinguished from pyramidal neurons based on their briefer action potential durations. In this study, we classified V1 cells into fast-spiking units (FSUs) and regular-spiking units (RSUs) and then examined spatial summation at high and low contrast. Our results revealed that the excitatory classical receptive field and the suppressive non-classical receptive field expanded at low contrast for both FSUs and RSUs, but the expansion was more marked for the RSUs than for the FSUs. For most V1 neurons, surround suppression varied as the contrast changed from high to low. However, FSUs exhibited no significant difference in the strength of suppression between high and low contrast, although the overall suppression decreased significantly at low contrast for the RSUs. Our results suggest that the modulation of spatial summation by stimulus contrast differs across populations of neurons in the cat primary visual cortex.

## Introduction

Most neocortical neurons are excitatory pyramidal neurons (70%-80% neoneurons), and the GABAergic ‘local circuit neurons’, which account for only 20–30% of cortical neurons, but show a rich morphological and electrophysiological diversity [1, 2]. In visual neocortical circuits, the interaction between excitatory and inhibitory neurons is a critical element in the shaping of receptive field structure [3–5] and in the maintaining stimulus selectivity [6–11]. Studies of neocortical circuits have shown that the connection patterns and functional properties of both neuron types have marked difference: excitatory pyramidal neurons can have both long-range and short-range connections, whereas inhibitory interneurons display more local connection patterns [1, 12]. Compared to excitatory pyramidal cells, inhibitory interneurons showed much broader tuning and tend to respond more strongly to sensory stimuli [13–14]. Recent studies have used the features of extracellular action potentials to characterize two physiological types of cortical neurons: regular-spiking units (RSUs) and fast-spiking units (FSUs). RSUs predominantly correspond to pyramidal neurons which have longer APs, whereas FSUs often correlate with parvalbumin-stained cortical cells and correspond to basket and chandelier cells [13–18].

In mammalian primary visual cortex, there is a larger non-classical receptive filed (nCRF) region beyond the classical receptive field (CRF), which does not respond directly to the visual stimulus but can suppress a cell's response [19, 20]. Several previous studies in primates and cats have reported that the extent of spatial summation in V1 depends on stimulus contrast. The excitatory CRF and suppressive nCRF usually expand as stimulus contrast decreases. Most V1 neurons show surround suppression at high and low contrast [21, 22]. Furthermore, Song et al. [23] have found that the different cell type might have contrast-dependent and contrast-independent spatial summation properties. Parvalbumin-expressed interneurons exhibit equivalent CRF sizes and similar surround suppression at high and low contrast [23]. However, in that study, they used 6 points to describe the spatial summation curve at high and low contrast. It is difficult to compare the effect of contrast on spatial summation between the excitatory pyramidal neurons and inhibitory interneurons at low spatial resolution. It is necessary to analyze the variation in spatial summation by using higher spatial resolution. Thus, in present study, our aim was to identify FSUs and RSUs using extracellular recording and then quantitatively examine the effect of contrast on spatial summation and surround suppression between the two cell types.

Our results showed that both FSUs and RSUs exhibited significant enlargement in CRF and nCRF size at low contrast, but the expansion was more marked for the RSUs than for the FSUs. For most V1 neurons, surround suppression varied as the contrast changed from high to low. However, FSUs exhibited no significant variation in the strength of suppression at different contrast, but the overall suppression decreased significantly at low contrast for the RSUs.

## Materials and Methods

### Animal Preparation and Maintenance

This study was carried out in strict accordance with the recommendations in the Guide for the Care and Use of Laboratory Animals of the National Institutes of Health. The protocol was specifically approved by the Committee on the Ethics of Animal Experiments of the Shanghai Institutes for Biological Sciences, Chinese Academy of Sciences (Permit Number: ERSIBS-621001C).

All surgery was performed under general anesthesia combined with local application of Lidocaine, and all efforts were made to minimize suffering. Detailed descriptions of the procedures for animal surgery, anesthesia and recording techniques can be found in a previous study [22]. Acute experiments were performed on 17 domestic cats (weighted 2.5–3.5 kg), cats were anesthetized prior to surgery with ketamine hydrochloride (30 mg/kg, I.V.), and then tracheal and venous cannulations were carried out. After surgery, the animal was placed in a stereotaxic frame to make a craniotomy and carry out neurophysiological procedures. During recording, anesthesia and paralysis were maintained with urethane (20 mg/kg/h) and gallamine triethiodide (10 mg/kg/h), respectively. Glucose (200 mg/kg/h) in Ringer's solution (3 ml/kg/h) was infused. Heart-rate, electrocardiography, electroencephalography (EEG), end-expiratory CO_2_ and rectal temperature were monitored continuously. Anesthesia was considered sufficient when the EEG indicated a permanent sleep-like state. Reflexes, including cornea, eyelid, and withdrawal reflexes, were tested at appropriate intervals. Additional urethane was immediately given I.V. when necessary. The nictitating membranes were retracted and the pupils dilated. Artificial pupils 3mm in diameter were used. Contact lenses and additional corrective lenses were applied to focus the retina on a screen for stimulus presentation. At the end of the experiment, the animal was sacrificed by an overdose of barbiturate I.V. (30 ml, 6%).

### Data Acquisition

Extracellular recordings were made from 154 neurons of the primary visual cortex of anesthetized cats using tungsten-in-glass microelectrodes with exposed tips of 5–10μm [24]. The electrode was advanced into the cortex using step-motor micro-drive (Narishige MO-91, Japan). The signal was amplified and band-pass filtered (0.3–10 kHz). Single-unit activity was amplified, converted to digital pulses and then recorded using a physiological instrument (Cerebus-128, Cyberkinetics, USA). Peri-stimulus time histograms (PSTHs) of unit responses were generated and analyzed on-line using custom-made software (written by MATLAB software). Spike waveforms were analyzed both during experiments and off-line, using standard software packages and customized software written specifically for this purpose.

### Visual Stimulus

Visual stimuli generated with a ViSaGe MKII visual stimulus generator (Cambridge Research Systems, UK), were presented to one eye using a computer display (MM906UT; IIYAMA, Japan; screen size: 40 cm × 30 cm). The monitor was placed 57 cm from the eyes. This visual stimulator could generate multiple patches of sinusoidal grating stimuli. Under computer control, the orientation, spatial and temporal frequency, and direction of movement of the gratings were matched to the preferred parameters of the cell under study and real-time analyses of the responses were performed. The contrast of the grating was 40% and the mean luminance was 10cd/m^2^. All measurements were made during the stimulation of the neuron’s dominant eye with the other eye occluded. All cells recorded were obtained from the area of the cortex representing the central 10° of the visual field. We first located the center of the CRF by placing a narrow patch of sine-wave grating patch at successive positions (in a random sequence) along the axes perpendicular or parallel to the optimal orientation of the cell and then measured the response to its drift. The center of the CRF was defined as the peak of the response profiles for both axes. All recorded cells had CRFs centered within 10° of the visual axis. Once the receptive field center was established, we performed size-tuning measurements. By measuring the neuronal response as a function of stimulus area, size-tuning curves were measured at two levels of contrast. The contrast levels were chosen from the linear region of the cell’s contrast-response function [22]. Low levels were set at that contrast at the contrast that generated a response that was 10% of the maximum. High contrasts were selected to elicit responses that were 90% of the saturation response for each cell. Each patch size was presented for 3–10 cycles (5 is in most cases) of the drifting grating and standard errors were calculated. Outside the grating patches, the screen remained at the same mean luminance as that for the stimulus patches (10cd/m^2^).

### Data Analysis

To quantitatively evaluate the contrast sensitivity of CRF responses, we fit the contrast-response response relationship using the following equation:

$$R=R_{\text{max}}\times C^{n}/(Cn+C_{50}^{n})$$ (1)

in which R is the neuronal response, C is the contrast of the periodic stimulus, and *R*_max_(maximal response), n (exponent of the power function, >0) and *C*_50_(semi-saturation contrast for one-half of *R*_max_) are free parameters. The hyperbolic ratio is commonly used in fitting the contrast-response relationship of the CRF response in the visual cortex because it provides a good description of this relationship [25].

**Fig 1:**
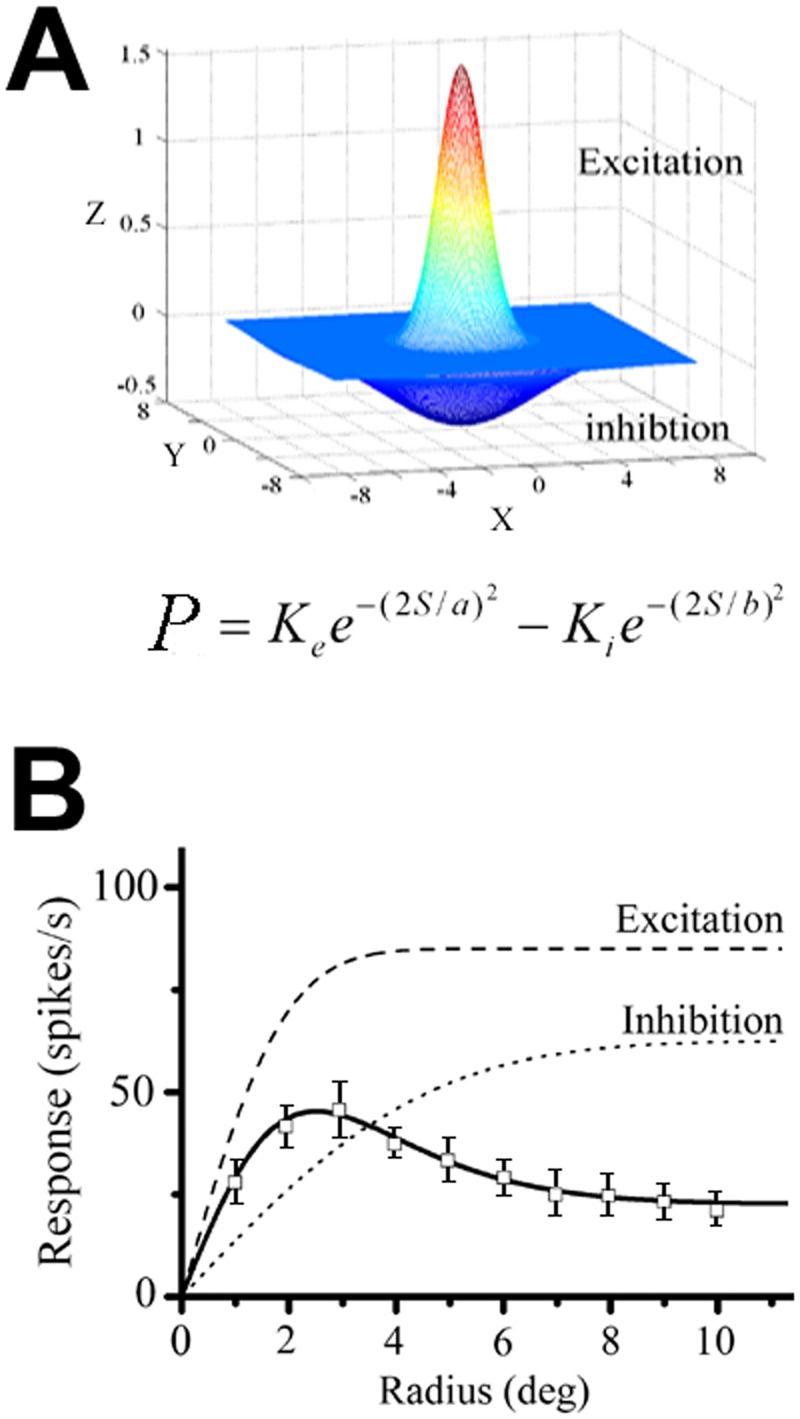
Difference of Gaussians (DOG) model. (A) The center-surround receptive filed organization is assumed to be fitted by two Gaussian curves: the narrower positive Gaussian representing the excitatory center or the CRF, while the broader negative Gaussian represents the inhibitory surround or the nCRF. K_e_ and a represent the strength and the space constants for the CRF, and K_i_ and b, those of the nCRF. The summation profile (R) of the model is represented as the difference of the two integrals of Gaussians. (B) A V1 neuron with suppressive summation. Dashed and dotted curves represent integrals of the excitatory and inhibitory components and the solid curve the linear combination of the two components that best fits the summation data.

The spatial summation curves for all recorded cells were fit using a difference of Gaussians (DOG) model [19]. In Fig 1, the two Gaussians are considered to be concentrically overlapping, and the summation profile can be represented as the difference of the integrals of the two Gaussians.

$$R(s)=R_{0}+K_{e}\int_{-s/2}^{s/2}e^{-{\left(2y/a\right)}^{2}}dy-K_{i}\int_{-s/2}^{s/2}e^{-{\left(2y/b\right)}^{2}}dy$$ (2)

where R_0_ is the spontaneous firing rate, and the first and second integral represent the relative contribution of the putative excitatory and inhibitory components, respectively. The excitatory Gaussian is described by its gain (K_e_), and by a space constant (a), and the inhibitory Gaussian by its gain (K_i_), and space constant (b). The variable *s* and *y* represent the radius of spatial summation.

A suppression index (SI) measure was estimated from the fitted curve, which was defined as follows:

$$SI=(R_{opt}-R_{asy})/(R_{opt}-R_{spon})$$ (3)

where R_opt_ is the maximum response, and R_asy_ is the asymptotic response. R_spon_ is the spontaneous firing level when no visual stimulus was given. When SI = 0, there is no suppression, and the response is either increasing or reaching a plateau. When SI = 1, the response is suppressed to the spontaneous firing level. In the majority of the cells, the SI was between 0 and 1.

All values were optimized to provide the least mean square error for the data. All fitting procedures were conducted with the MATLAB optimization toolbox, using the CONSTR and FMINCON nonlinear least-squares functions. To evaluate how well our experimental data fit the model, the goodness of each fit (GEF) was established by calculating the mean fraction error, which was defined as

$$E=\frac{1}{N}\sum_{j=1}^{N}\frac{{\left(theory_{j}-data_{j}\right)}^{2}}{{\left(\frac{1}{N}\sum_{k=1}^{N}theory_{k}\right)}^{2}}$$ (4)

where theory_j_ and data_j_ are the expected response theory and the experimental response data to the jth stimulus size, respectively and theory_k_ are the expected response theory to the kth stimulus size.

All population values given below are expressed as the mean plus or minus the standard error of the mean. All two-way comparisons were tested for significance with the Mann-Whitney U test.

## Results

### Classification of cortical cells based on extracellular recording

**Fig 2:**
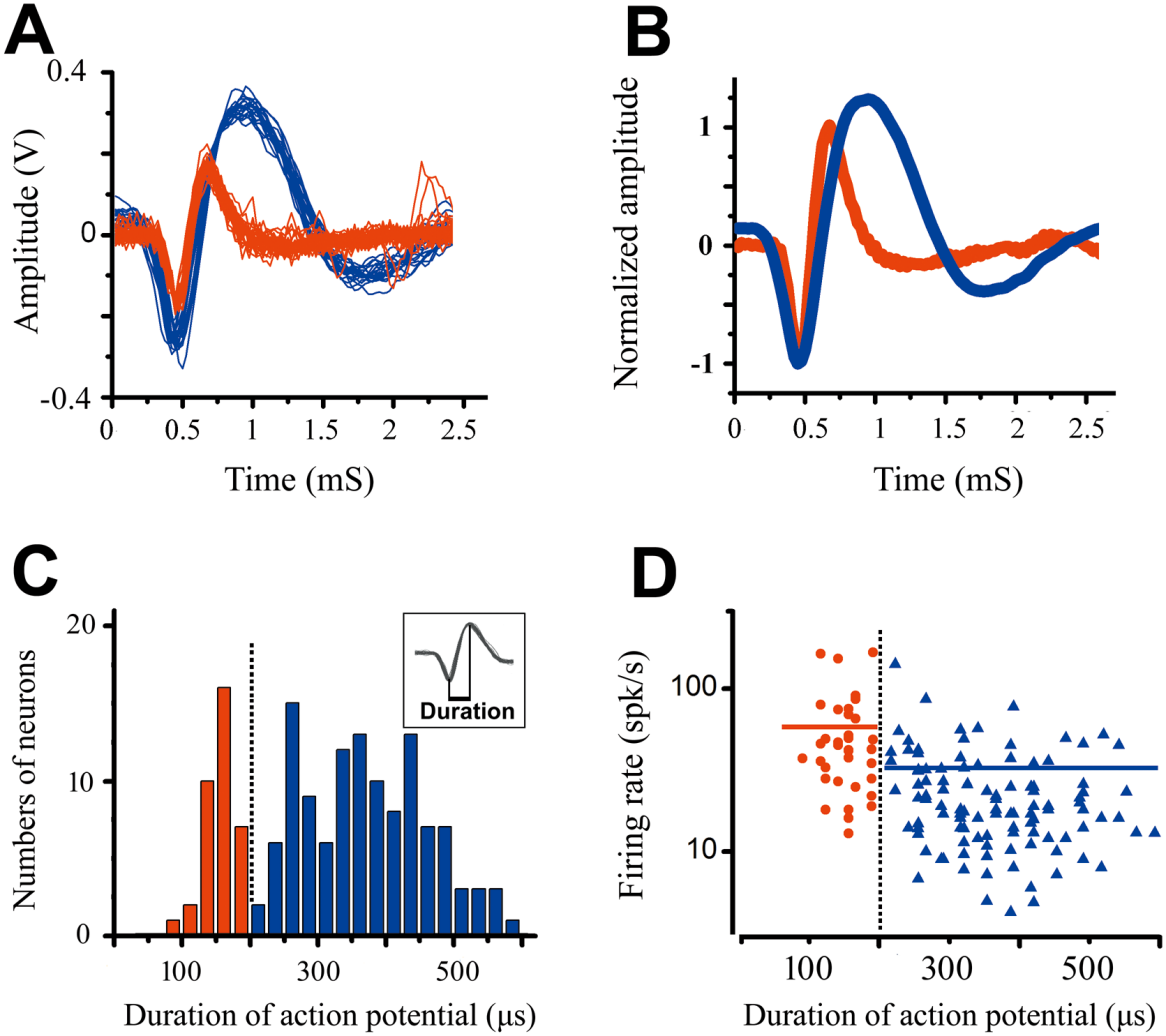
Classification of the FS-Units and RS-Units. (A) Shown were representative traces, recording from two neurons in V1 cat. (B) The recorded action potential waveforms were averaged and normalized. (C) The distribution of waveform durations was significant bimodal according to Hartigan’s dip test (P < 0.01). (D) The scatterplots of each cell’s peak firing rate versus spike duration. The average of peak firing rates for FSUs was significant stronger than those of RSUs (P = 0.0003). The blue and red solid line indicate the average of peak firing rate of RSUs and FSUs for each.

Single-unit recordings were made in the V1 of anaesthetized adult cat. We recorded spatial summation from 154 single neurons across all cortical layers in cat V1. All of the neurons showed a typical extracellular waveform, with a negative deflection followed by a positive deflection. We analyzed the spike waveform of recorded neurons to classify these cells as putative pyramidal neurons or as interneurons. For each well-isolated unit, a number of action potentials (≥20 spikes) were collected. Then they were aligned by their troughs and averaged (Fig 2A and 2B). The SWD (spike width duration) was calculated from the average waveform as the time between the maximum and minimum values of the waveform. Fig 2C shows the histogram of SWD for 154 neurons. Consistent with previous reports, there were two peaks in the histogram of action potential duration: one at 160μs and the other at approximately 350μs. According to Hartigan’s dip test, the distribution of SWD was significantly bimodal (*P* < 0.01). We classified neurons as interneurons if their SWD was ≤200μs and will refer to them as FSUs. Neurons were classified as pyramidal neurons if their SWD was >200μs and are referred to as RSUs in the following analysis. With this classification, 36 FSUs and 118 RSUs were isolated from our recordings. The average action potential duration was 160.3 ± 26.1 μs for the FSUs and 371.2 ± 94.4 μs for the RSUs, respectively. Fig 2D shows a scatterplot of each cell’s peak firing rate versus SWD. We compared each cell’s optimal response between the two categories when grating orientation, spatial and temporal frequency, movement direction, and size were set as the preferred parameters of each cell. The peak firing rate was 52.9 ± 39.5 spikes/s for FSUs, which was significantly greater than the rate for RSUs (32.4 ± 25.1 spikes/s, P = 0.0003, Mann-Whitney *U* test). These results are consistent with earlier findings using the same classification scheme [13, 17–18], which show that FSUs tend to exhibit higher activity than RSUs under the same stimulation.

### Contrast-dependent Variance in CRF and nCRF between FSUs and RSUs

**Fig 3:**
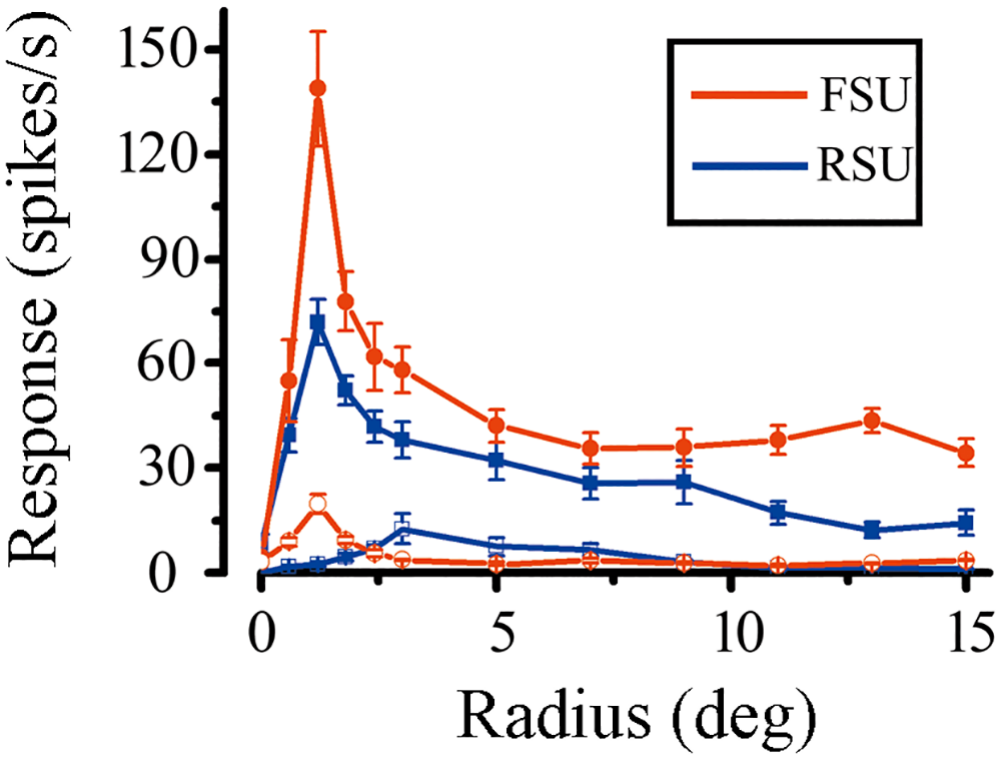
The spatial summation curves of two V1 neurons at high and low contrast. The red and blue line represents a FSU and a RSU for each. The curve with solid symbols represents the spatial summation curve measured at high contrast and the curve with open symbols at low contrast.

The receptive field size is partly dependent on the eccentricity of their location in the visual field. All cells recorded in present study were obtained from the area of the cortex representing the central 10° of the visual field. We found no significant difference in average of retinal eccentricity between the RSUs and FSUs (P > 0.05). Using circular patches of drifting sinusoidal gratings centered at the middle of the CRF, we determined the spatial summation curves of 154 V1 cells at high and low contrast. We wanted to investigate the variation in spatial summation and surround suppression when the stimulus contrast was changed. In present study, a cell was excluded either if it had no surround suppression (SI < 0.1) or its GEF in spatial summation curve was larger than 0.2. For the entire population, 2 FSUs and 38 RSUs were excluded because of no surround suppression at high contrast, and the other 4 RSUs were also excluded due to large GEF. Thirty-four FSUs and 76 RSUs were further investigated for the variation in spatial summation and surround suppression. The GEF of the 110 V1 neurons ranged from 0.003 to 0.12, with a mean GEF of 0.022 across the population. Fig 3 shows example spatial summation curves for each category at high and low contrast, separately. From the fitted curves, the CRF size corresponds to the excitatory space constant ‘a’ and the nCRF size corresponds to the suppressive space constants ‘b’.

**Fig 4:**
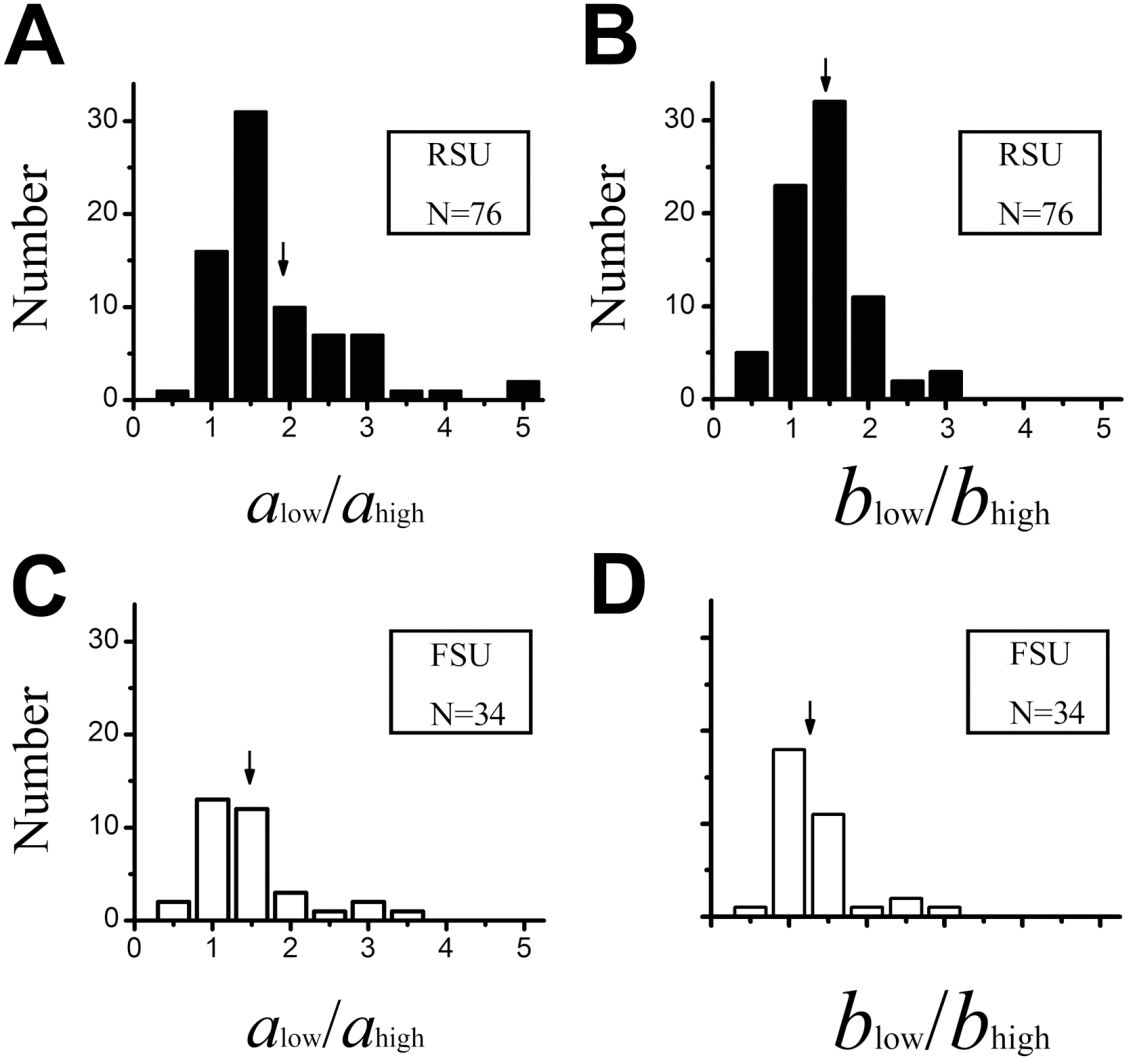
Histograms of contrast-dependent variations in the excitatory and inhibitory spatial spread for RSUs and FSUs. The ratio a_low_/a_high_ was used to describe the enlargement in the extent of CRF and b_low_/b_high_ for nCRF. The solid and hollow columns represent RSUs and FSUs, respectively. The arrows indicate the average in each subgraph.

We measured the average CRF and nCRF size (diameter of stimulus grating) for each category at high and low contrast. For the FSUs, CRF size was 3.7 ± 2.4deg, and nCRF size was 10.8 ± 4.9deg at high contrast, whereas CRF size was 5.1 ± 3.0deg, and nCRF size was 13.4 ± 5.4deg at low contrast. However, for the RSU population, CRF size was 3.1 ± 1.5deg and nCRF size was 9.6 ± 4.6deg at high contrast; while the CRF size was 5.2 ± 2.6 deg, and nCRF size was 12.9 ± 5.5deg at low contrast. We then compared the contrast-dependent variation in spatial summation for the FSUs and RSUs. The ratio a_low_/a_high_ was used to describe the variation in the extent of CRF and the ratio b_low_/b_high_, for describing the variation in the extent of nCRF. The greater the ratio, the greater the enlargement of the spatial extent. Fig 4 shows the distribution of the ratio a_low_/a_high_ versus b_low_/b_high_ ratio for the FSUs and RSUs. In the population of FSUs, the mean enlargement was 1.49 ± 0.71 times for the CRF and 1.32 ± 0.50 times for the nCRF. In contrast, the average of enlargement was 1.83 ± 0.88 times for CRFs and 1.45 ± 0.49 times for the nCRFs in the RSUs. There were significant differences in CRF and nCRF enlargement as contrast was changed from high to low between the FSU and RSU populations (CRF: *P* = 0.0061; nCRF: *P* = 0.0169; Mann-Whitney *U* test).

### Strength of surround suppression for FSUs and RSUs

**Fig 5:**
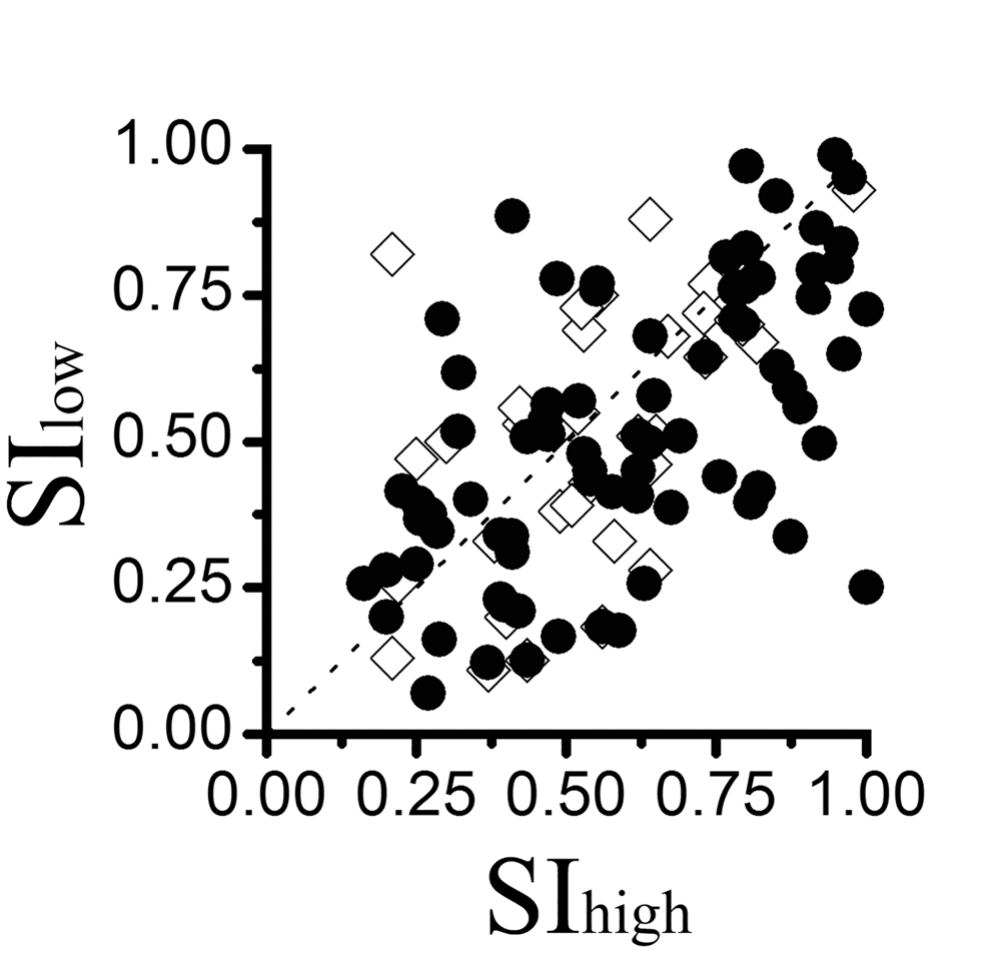
The scatter plot of the estimates of surround suppression at high contrast versus low contrast. The solid circle and hollow diamond indicate RSUs and FSUs, respectively.

The strength of surround suppression can be represented by a suppression index, SI (see methods). An SI of 1.0 indicates the cell’s response is completely abolished by surround suppression and an index of 0 indicates the response of the cell grew to a maximal asymptote. We estimated the contrast-dependent variation in the strength of surround suppression in RSUs and FSUs. In Fig 5, suppression index (SI) estimates of FSUs and RSUs are compared for low versus high stimulus contrast. For FSUs, the mean value of SI was 0.54 ± 0.19 at high contrast and 0.51 ± 0.23 at low contrast (*P* >0.05, Mann-Whitney *U* test). Nevertheless, the average SI was 0.60 ± 0.24 at high contrast and 0.51 ± 0.23 at low contrast for the RSUs (*P* <0.05, Mann-Whitney *U* test).

**Fig 6:**
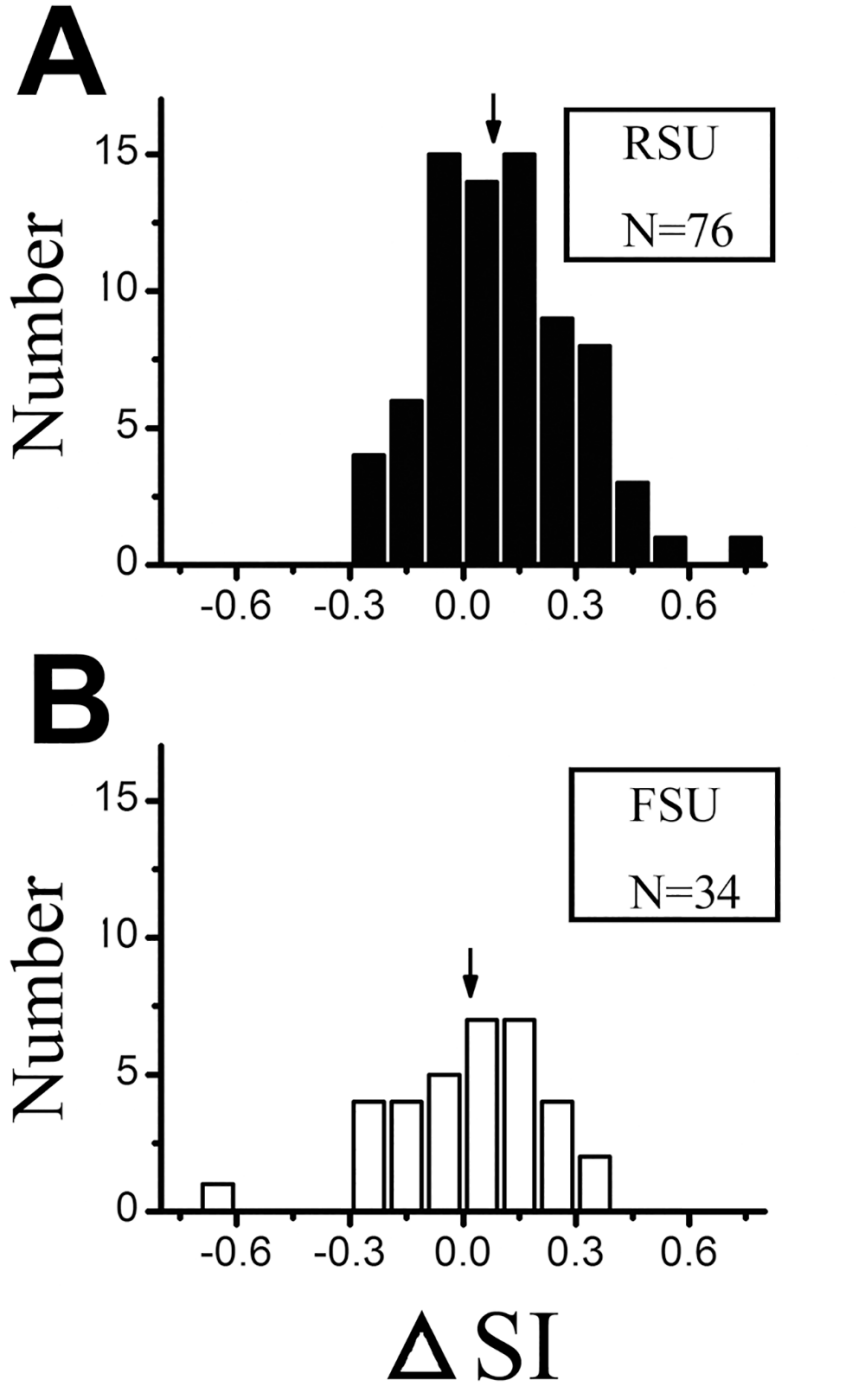
The histogram of changes in strength of surround suppression for FSUs and RSUs. The △SI indicate the difference between SI_high_ and SI_low_. The arrows indicate the average, which was 0.02 for FSUs and 0.08 for RS-Units (P >0.05).

We further analyzed the △SI, which represents the difference in SI at high contrast and low contrast (△SI = SI_high_-SI_low_). Positive values of △SI indicate more surround suppression at high contrast, whereas negative values indicate less surround suppression at high contrast. Fig 6 shows the histogram of △SI across the FSU and RSU populations. The average △SI was 0.02 ± 0.20 for FSUs and 0.08 ± 0.21 for RSUs; However, there was no significant difference between the FSUs and RSUs (*P* >0.05, Mann-Whitney *U* test).

## Discussion

Our results show that neurons in cat V1 can be divided into FSUs and RSUs based on their action potentials waveform. The average firing rates of FSUs nearly two-folds than that of RSUs, when stimulating the receptive field with optimal grating in the receptive field. Stimulus contrast has significant effect on the spatial summation of both FSUs and RSUs. However, the effect is more marked for the RSUs than for the FSUs.

### Relationship between cells type and action potential waveform

Previous investigations, which have combined intra- and extracellular recordings, have established the classification of FSUs and RSUs, according to the results both in slice and in vivo [26–29]. Other reports on neurophysiology showed that most pyramidal neurons (70%-80%) and part of interneuron (10%-15%) have broad action potentials. The remaining GABAergic interneuron (10%-15%), with the morphology of basket cells and chandelier cells, are fast-spiking units, with the narrow action potentials [30–38]. The narrow action potentials recorded from fast-spiking cells are due to the high levels of expression of two classes of potassium channels [39]. Spike-waveform-based identification of FSUs and RSUs have been described in several species and cortical areas on recent studies, including the hippocampus and neocortex of the rat [40, 41], monkey [13, 37], and cat [16, 17]. Intracellular recordings and morphological reconstructions are required for an exactly identification, and thus some degree of classification error may be inevitable. Our results apply to the parvalbumin-expressing inhibitory interneurons, which comprise a subset of neocortical inhibitory interneuron [1, 35, 42]. Some pyramidal cells may also have narrow action potential that may create classification error [43, 44]. But we conclude that the majority of RSUs in our studies are pyramidal neurons, possibly intermixed with a small number of regular-spiking interneurons, and that our samples of narrow action potentials largely correspond to fast-spiking inhibitory interneurons with the morphology of basket cells and chandelier cells.

### Different neuronal response properties and cognitive function between FSUs and RSUs in visual cortex

Our study showed the enlargement of CRF and nCRF at low contrast was significantly different between FSUs and RSUs. In the study of Song and Li [23], they subdivided V1 neurons into two major groups, contrast-dependent cells and contrast-independent neurons. They found the pyramidal cells had a significant increase in CRF size and inhibitory interneuron exhibited constant CRF at 80% and 10% contrast. However, our result revealed the inhibitory interneurons still showed contrast-dependent spatial summation. We supposed the differences are due to the following reasons. First, they choose the high contrast at 80% and low contrast at 10% but we choose the high and low contrast by the cell’s contrast response function. There are some arbitrary to choose the high and low contrast as constant value. Second, they just used 6 points to describe the in spatial summation curves, but more than 10 points were used in our investigation to make the results more accurate.

Many investigations discovered the inhibitory interneurons have distinctive response property and play an important role in visual cortex. In primary visual cortex, putative inhibitory neurons generally have less selective, and nonlinear, responses than pyramidal neurons [45–50]. Many anatomical and physiological studies in neuronal circuit have shown fast spiking neurons play an important role in mediating inhibition in local cortical circuits [51, 52]. They receive strong excitatory input from layer 4 and provide feedforward inhibition to neighboring layer 2/3 pyramidal cells [53, 54]. Paired recordings also shown fast-spiking neurons can receive inputs from pyramidal neurons and generate feedback inhibition in the same cells that excites themselves [55]. In this study, we have found the difference in contrast-dependent effect on visual spatial summation between FSUs and RSUs. RSUs are mainly functional neurons in cerebral cortex. Shrinkage of the excitatory CRF and increase in surround suppression may result in an improvement in spatial resolution of visual detection and capacity to precisely localize features of an image. At low contrast, expansion of spatial summation and a decrease in surround suppression produce increased sensitivity and a better detection capability for weak signals. The FSUs are regulatory neurons, which can suppress the neuronal circuit accurately to perform the cognition function. But they have less contrast-dependent spatial summation.

### Neurophysiology bases of contrast effect on spatial summation

Substantial evidence have accumulated indicating that geniculocortical feedforward, intra-cortical connections within V1 [56–57] and extrastriate feedback [57, 58] play different roles in visual spatial summation of V1 neurons. In previous studies, the excitatory center of V1 can be separated into a_high_ and a_low_, which were measured by the spatial summation curves at high and low contrast respectively. The a_low_ was approximately twice as the a_high_. The far surround is the region outside the a_low_ over which presentation of stimuli at the same orientation as the center stimulus usually suppresses the response of the cell to optimally oriented gratings in the center. The geniculocortical feedforward afferents to V1 mainly integrate signals within the a_high_. Intra-areal horizontal connections, which play an important role in shaping the contrast-dependent spatial summation of V1 neurons, extend beyond the a_high_, and are commensurate with the a_low_. Horizontal axons do not drive their target neurons, but only elicit subthreshold response [59]. Both excitatory pyramidal cells and inhibitory interneurons have same chance to receive the inputs from the horizontal fibers, which could have disynaptic inhibitory effects as well as direct excitatory actions [59, 60].

Our findings showed the enlargement of CRF and nCRF at low contrast was significantly different between FSUs and RSUs. Several previous studies provide a possible explanation for the observed expansions of the size of V1 receptive fields when contrast decreases [61–63]. The excitatory pyramidal neurons are easy to be active at low contrast, but the response will be increase gradually as the contrast is increase. In contrast, the inhibitory interneurons are essentially silent at low contrast, but the activity of local inhibitory neurons will rapidly increase at high contrast. The high response threshold of the inhibitory interneurons will result the subthreshold excitatory from horizontal interaction at low contrast, which are difficult to activate the center interneuron. Once the center interneuron was activated at a higher contrast, most local interneurons also have been activated. Thus, the spatial summation of interneurons may show less-dependent on stimulus contrast.
